# A simple method for HPLC retention time prediction: linear calibration using two reference substances

**DOI:** 10.1186/s13020-017-0137-x

**Published:** 2017-06-19

**Authors:** Lei Sun, Hong-yu Jin, Run-tao Tian, Ming-juan Wang, Li-na Liu, Liu-ping Ye, Tian-tian Zuo, Shuang-cheng Ma

**Affiliations:** 1 National Institutes for Food and Drug Control, No. 2 Tiantan Xili, Dongcheng District, Beijing, 100050 People’s Republic of China; 2 Xinjiang Institute for Food and Drug Control, Urumqi, 830004 China; 3 Chemmind Technologies Co., Ltd., Beijing, 100085 China; 4 Huainan Food and Drug Inspection Center, Huainan, 232007 China

**Keywords:** RP-HPLC, Retention time, Relative retention, Linear calibration using two reference substances, Multi-component analysis, Traditional Chinese medicines

## Abstract

**Background:**

Analysis of related substances in pharmaceutical chemicals and multi-components in traditional Chinese medicines needs bulk of reference substances to identify the chromatographic peaks accurately. But the reference substances are costly. Thus, the relative retention (RR) method has been widely adopted in pharmacopoeias and literatures for characterizing HPLC behaviors of those reference substances unavailable. The problem is it is difficult to reproduce the RR on different columns due to the error between measured retention time (t_R_) and predicted t_R_ in some cases. Therefore, it is useful to develop an alternative and simple method for prediction of t_R_ accurately.

**Methods:**

In the present study, based on the thermodynamic theory of HPLC, a method named linear calibration using two reference substances (LCTRS) was proposed. The method includes three steps, procedure of two points prediction, procedure of validation by multiple points regression and sequential matching. The t_R_ of compounds on a HPLC column can be calculated by standard retention time and linear relationship.

**Results:**

The method was validated in two medicines on 30 columns.

**Conclusion:**

It was demonstrated that, LCTRS method is simple, but more accurate and more robust on different HPLC columns than RR method. Hence quality standards using LCTRS method are easy to reproduce in different laboratories with lower cost of reference substances.

**Electronic supplementary material:**

The online version of this article (doi:10.1186/s13020-017-0137-x) contains supplementary material, which is available to authorized users.

## Background

Multi-components analysis is an effective strategy for quality control of traditional Chinese medicines (TCMs), which have complex chemical profiles. But the classic external standard method was severely confined in its application due to the high cost of reference substances. As a consequence, substitute reference substance methods such as extractive reference substance (ERS) method and single standard to determine multi-components (SSDMC) method for overall quality control of TCMs have emerged, and widely used in Chinese pharmacopoeia 2015 edition, the United States Pharmacopoeia (USP39-NF34) and literatures [1–10]. In general, ERS method provides only one reference chromatogram in the pharmacopoeias, instructions of ERS and literatures. But there are hundreds of brands of C_18_ columns in the market. It means that the reference chromatogram may be different from the actual chromatogram. Due to the column types and other various factors, the error between measured retention time (t_R_) and predicted t_R_ by the relative retention (RR) method cannot be ignored sometimes.

In order to improve the reproducibility of chromatographic separation and RR, the method of classification of C_18_ columns has been proposed [11–15]. The columns were divided into three types: A, B and EP. Although the same type of columns was used to repeat the analytical methods, the differences in the performance and the separation effects were still large. And then the methods for selecting columns with equivalent selectivity, such as the USP approach [16], the PQRI approach [17, 18] and Katholieke Universiteit Leuven column classification system [19–21] were proposed. Take PQRI approach [17, 18] as an example, hydrophobicity (H), steric interaction (S), hydrogen-bond acidity (A), hydrogen-bond basicity (B) and ion-exchange capacity (C), were used to describe the performance of the column. And the similarity between a column and the reference column was calculated by these five parameters. When the similarity was less than three, the two columns were regarded to be equivalent. Using the equivalent column, the reproducibility of separation and RR could be improved to some extent. However, in addition to column, many other factors also have great influences on the chromatograms, such as the dead volume of chromatographic system, the different structure of analytes, the complexity of the chromatographic conditions, and so on. Therefore, it is necessary to develop a method that takes all aforementioned factors into account to reduce the prediction error of the t_R_.

According to the thermodynamic theory of liquid chromatography, there is a linear relationship between the t_R_ of the compounds on two different HPLC systems (including chromatographs and columns) [22]. For better understanding, the pdf of reference 22 (in Chinese, Additional file 1) and the English version of reference 22 (only the section of theory was translated, Additional file 2) are provided. Combined with the above principle and previous studies [23–25], a novel method using two reference substances for predicting HPLC t_R_ has been proposed (linear calibration by two reference substances, LCTRS). The St_R_ (arithmetic average of t_R_ for the same compound on different HPLC system under the same chromatographic conditions) is used as the reference value, and the linear regression is used as the basic algorithm for t_R_ prediction. In this study, the method was validated in two medicines on 30 C_18_ columns. Compared with the RR method, LCTRS method is proved to be more accurate, and more robust on different HPLC columns. Hence, it provides a good prospective application in quantification of multi-components in TCMs as well as related substances in pharmaceutical chemicals.

## Methods

The Minimum Standards of Reporting Checklist contains details of the experimental design, and statistics, and resources used in this study (Additional file 3).

### Instruments and reagents

Waters e2695 HPLC (2998PDA detector), Agilent 1260 HPLC (DAD detector), and Shimadzu LC-2010A HT HPLC (UV–Vis detector) were used. Matlab software was provided by Math Works Inc. USA. 30 C_18_ columns (shown in Table 1), from 13 manufacturers, included A, B, and EP types were used. And most columns belong to type B according to the previous study [11–15]. According to the PQRI approach [17, 18] and using the data from the USP website (http://www.usp.org/USPNF/columns.html), the similarity of columns were calculated using col1 as the reference column. The similarity (0–13.18) showed that the differences among the columns were large, which indicated that the selected columns are in a wide range and have good representative trait.

**Table 1:** t_R_ (min) of four saponins in Paridis on different columns

No.	Brand	Chonglou saponin VII	Chonglou saponin VI	Chonglou saponin II	Chonglou saponin I
col1	Discovery C_18_^a^	21.234 ± 0.021	23.001 ± 0.006	32.773 ± 0.007	35.118 ± 0.004
col2	Discovery C_18_^b^	16.555 ± 0.010	17.989 ± 0.006	27.161 ± 0.011	29.154 ± 0.002
col3	Xbridge C_18_^a^	21.101 ± 0.004	23.070 ± 0.027	32.483 ± 0.021	34.963 ± 0.006
col4	BDS HypersilC_18_^a^	21.014 ± 0.011	22.898 ± 0.018	32.679 ± 0.032	35.170 ± 0.036
col5	Inertsil ODS-2^a^	22.132 ± 0.009	24.502 ± 0.004	33.176 ± 0.017	35.936 ± 0.003
col6	Kromasil C_18_^a^	21.276 ± 0.012	23.693 ± 0.016	32.618 ± 0.006	35.929 ± 0.007
col7	Luna C_18_(2)^a^	20.760 ± 0.018	23.362 ± 0.008	30.941 ± 0.011	33.813 ± 0.003
col8	Luna C_18_(2)^b^	16.551 ± 0.008	18.865 ± 0.023	25.923 ± 0.016	28.310 ± 0.011
col9	Inertsil ODS-3^b^	17.225 ± 0.006	19.640 ± 0.005	26.754 ± 0.013	29.471 ± 0.005
col10	Alltima C_18_^a^	20.856 ± 0.008	23.752 ± 0.021	31.687 ± 0.011	34.872 ± 0.004
col11	Symmetry C_18_^a^	21.016 ± 0.015	22.076 ± 0.016	32.470 ± 0.023	35.476 ± 0.010
col12	Gemini C_18_^a^	21.300 ± 0.014	23.756 ± 0.007	31.599 ± 0.003	34.337 ± 0.015
col13	CapcellpakC_18_MG^a^	21.076 ± 0.012	23.828 ± 0.004	31.627 ± 0.006	34.695 ± 0.001
col14	Zorbax Extend-C_18_^a^	17.201 ± 0.022	19.731 ± 0.003	27.525 ± 0.009	30.504 ± 0.005
col15	Sunfire C_18_^a^	21.652 ± 0.013	24.065 ± 0.024	32.375 ± 0.010	35.197 ± 0.006
col16	Sunfire C_18_^b^	17.501 ± 0.006	19.571 ± 0.018	27.401 ± 0.004	29.826 ± 0.010
col17	Nucleosil C_18_ HD^a^	21.452 ± 0.013	23.735 ± 0.006	32.747 ± 0.019	35.513 ± 0.011
col18	ODS Hypersil^a^	19.669 ± 0.010	21.583 ± 0.022	30.361 ± 0.007	32.657 ± 0.005
col19	CapcellpakC_18_AQ^a^	20.092 ± 0.013	22.560 ± 0.010	29.568 ± 0.006	32.218 ± 0.005
col20	Spherisorb ODS2^a^	18.684 ± 0.006	21.334 ± 0.021	28.580 ± 0.008	31.385 ± 0.004
col21	Zorbax SB-C_18_^a^	18.438 ± 0.015	20.986 ± 0.010	28.085 ± 0.008	30.849 ± 0.007
col22	DiamonsilC_18_^a^	22.082 ± 0.021	25.078 ± 0.005	32.475 ± 0.020	35.721 ± 0.023
col23	DiamonsilC_18_^b^	16.769 ± 0.007	19.154 ± 0.008	26.240 ± 0.011	28.935 ± 0.004
col24	Diamonsil C_18_(2)^a^	19.296 ± 0.016	22.819 ± 0.004	31.192 ± 0.019	34.710 ± 0.009
col25	Kromasil Eternity C_18_^a^	19.732 ± 0.017	22.099 ± 0.018	29.535 ± 0.004	32.153 ± 0.021
col26	Shim-pack VP-ODS^b^	19.015 ± 0.007	21.059 ± 0.014	29.401 ± 0.002	31.845 ± 0.001
col27	Agilent HC-C_18_^a^	23.492 ± 0.247	25.839 ± 0.250	34.884 ± 0.344	37.637 ± 0.330
col28	Agilent TC-C_18_^a^	20.609 ± 0.003	22.574 ± 0.005	30.806 ± 0.003	32.783 ± 0.002
col29	Venusil MP C_18_^b^	17.970 ± 0.007	20.468 ± 0.011	27.287 ± 0.031	29.985 ± 0.007
col30	Nucleosil C_18_ AB^a^	18.348 ± 0.012	20.209 ± 0.017	29.233 ± 0.011	31.893 ± 0.014
	St_R_ (Average t_R_)	19.803	22.110	30.319	33.035

^a^4.6 mm × 250 mm × 5 μm

^b^4.6 mm × 150 mm × 5 μm

Reference substances of psoralen, isopsoralen, Chonglou saponin I, Chonglou saponin II, Chonglou saponin VI, Chonglou saponin VII, ethinylestradiol, and herbal reference substances including Psoraleae Fructus (Psoraleae) and Paridis Rhizome (Paridis), were supplied by the National Institutes for Food and Drug Control, China. Methanol, acetonitrile, and phosphoric acid were HPLC graded and supplied by the Fischer Company, USA. Ammonium nitrate (analytical grade) was supplied by Beijing Chemical Works. Water was prepared by Milli-Q system, Millipore Company, USA.

### Preparation of sample solution

Psoraleae [26]: weigh 0.5 g of the powder and place it in a 50-mL stopper conical flask, then add 25 mL of ethanol. Sonicate the mixture for 30 min and centrifuge for 5 min. Filter the supernatant through a 0.45-μm PTFE filter.

Paridis [27]: weigh 0.5 g of the powder to a stopper conical flask, add 25 mL of ethanol, heat under reflux on a water bath for 30 min, cool and filter the supernatant through a 0.45-μm PTFE filter.

### Chromatographic conditions

Psoraleae [26]: mobile phase A was water and mobile phase B was methanol. The elution procedure was shown below: 0–20 min, 50%B→70%B; 20–45 min, 70%B→85%B; 45–50 min, 85%B→90%B; 50–60 min, 90%B. Detection wavelength was 308 nm. Paridis [27]: mobile phase A was water and mobile phase B was acetonitrile. The elution procedure was shown below: 0–40 min, 30%B→60%B; 40–50 min, 60%B→30%B; 50–60 min, 30%B. Detection wavelength was 203 nm. Column temperatures were both set at 30 °C and flow rate was 1.0 mL/min.

## Results

### HPLC chromatogram of samples

**Fig. 1:**
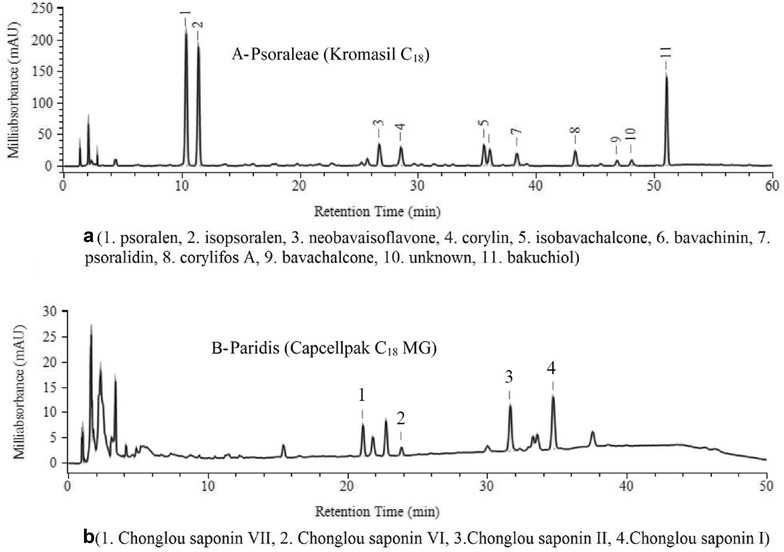
HPLC chromatograms of samples

The typical chromatograms of Psoraleae and Paridis were shown in Fig. 1. The peaks were mainly identified by the reference substances. For those peaks without reference substances, UV–Vis spectrum and mass spectrum were used for identification.

### Standard retention time (St_R_)

Under the same chromatographic conditions, measured retention time (t_R_mea) of the four saponins in Paridis on different chromatographic systems (which includes HPLC instruments and columns, hereinafter referred to as columns due to the differences of t_R_ mainly caused by columns) were shown in Table 1. The arithmetic average of t_R_ for the same compound on different columns is called St_R_, formula (1). Just like RR, St_R_ is the reference value for calculating the predicted retention time (t_R_pre) of analyte in the samples. Theoretically, under the same chromatographic condition, the RR calculated by different columns is constants, but St_R_ is not. It will be discussed in Section "Minimum number of columns for St_R_ calculation" that the advantages of using St_R_ was better than t_R_ of any single column. In this paper, the deviation (Δt_R_) of t_R_mea and t_R_pre (formula 2) was used to evaluate the merits and defects of RR method and LCTRS method.

1 $$ St_{R} = \mathop \sum \limits_{i = 1}^{n} t_{Ri} /n \quad (n \ge 1) $$

2 $$ \Delta t_{R} = \left| {t_{R} mea - t_{R} pre} \right| $$

### Linear principle of LCTRS

**Fig. 2:**
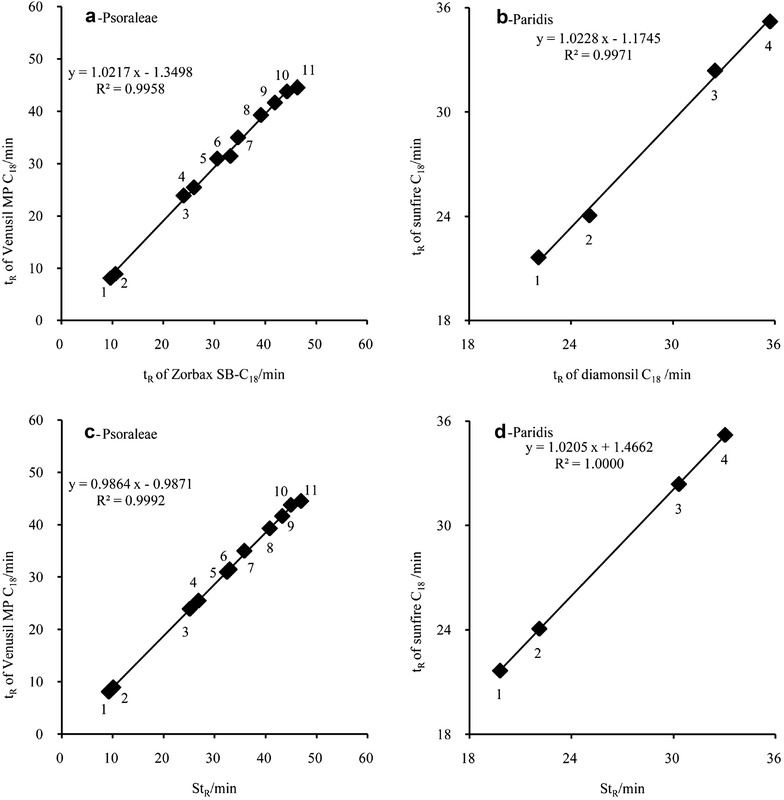
Linear fitting results of Psoraleae and Paridis, code No. is the same as that in Fig. 1

According to the chromatographic thermodynamic theory, Wang et al. proved that there was a linear relationship between the t_R_ of the same compounds on different HPLC system (mainly considered as columns) under the same chromatographic conditions [22], as expressed in formula (3) and Fig. 2a, b.

3 $$ t_{R} coli = a \times t_{R} colj + b $$

Since formulas (1) and (3) are both linear, thus there is a linear relationship between t_R_ and St_R_ for each compound, as shown in formula (4) and Fig. 2c, d. It is noteworthy that the correlation coefficient of the linear regression is higher than that shown in Fig. 2a, b.

4 $$ t_{R} coli = a \times St_{R} + b $$

### Minimum number of columns for St_R_ calculation

**Fig. 3:**
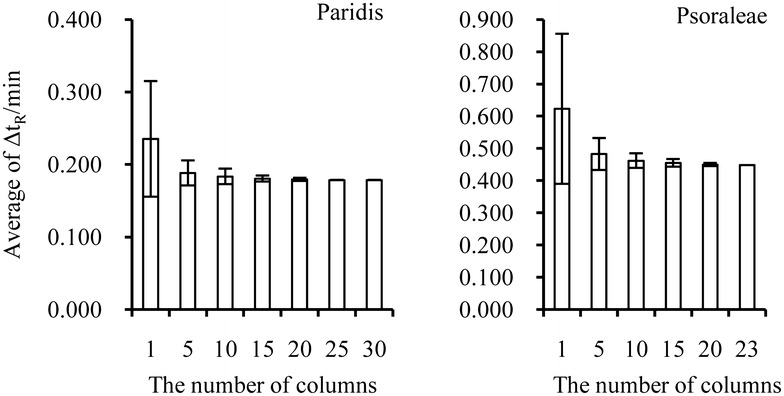
Prediction results of different number of columns for S_tR_ calculation

Theoretically, t_R_ on any column can be used as reference value for linear fitting. But the Δt_R_ calculated with random column were instable. Thus, the reasonable number of columns for St_R_ calculation was thoroughly investigated by random sampling. St_R_ was calculated based on 1, 5, 10, 15, 20, 25, and 30 columns combined with non-replicate random sampling times of 30, 100, 100, 100, 100, 100, and 1, respectively. The value of St_R_ with t_R_mea on 30 columns was used to fit multiple point linear equation. The averages of Δt_R_ (average ± standard deviation) were calculated, as shown in Fig. 3. For both medicines, the prediction deviation was reduced with increasing number of columns. However, the prediction accuracy will not be significantly improved when the number of columns reaches five, which is considered as a low-cost and reasonable limit. It is recommended to choose five–fifteen columns for St_R_ calculation.

Even for the columns with same type of packing material, there are still some differences among the column stationary phase, packing techniques and errors in the process of chromatographic analysis. Those differences will cause deviation of t_R_. The physical explanation of St_R_ calculation was to evenly mix and refill the stationary phase of the columns selected. Because of reducing the random and system errors, the prediction result was accurate and robust.

### Procedure of two points prediction

For RR method, only one compound was chosen as reference compound (reference substance required), and RR of all other compounds were used as reference value for calculating t_R_pre. For LCTRS method, two compounds were chosen as reference compounds (reference substances required), and St_R_ of all other compounds were used as reference value for calculating t_R_pre. The reference compounds, the value of RR and St_R_ were shown in Tables 2 and 3.

**Table 2:** RR and reference compound for RR method

	Peak 1	Peak 2	Peak 3	Peak 4	Peak 5	Peak 6	Peak 7	Peak 8	Peak 9	Peak 10	Peak 11
Psoraleae (n = 23)	0.226	0.247	0.615	0.657	0.794	0.808	0.878	1.000^a^	1.061	1.103	1.152
Paridis (n = 30)	0.652	0.729	1.000^a^	1.090							

^a^Reference compound

**Table 3:** St_R_ (min) and reference compound for LCTRS method

	Peak 1	Peak 2	Peak 3	Peak 4	Peak 5	Peak 6	Peak 7	Peak 8	Peak 9	Peak 10	Peak 11
Psoraleae (n = 23)	9.271	10.122^a^	25.143	26.854	32.437	32.951	35.843	40.793^a^	43.254	44.950	46.937
Paridis (n = 30)	19.803^a^	22.156	30.319	33.035^a^							

^a^Reference compound

**Fig. 4:**
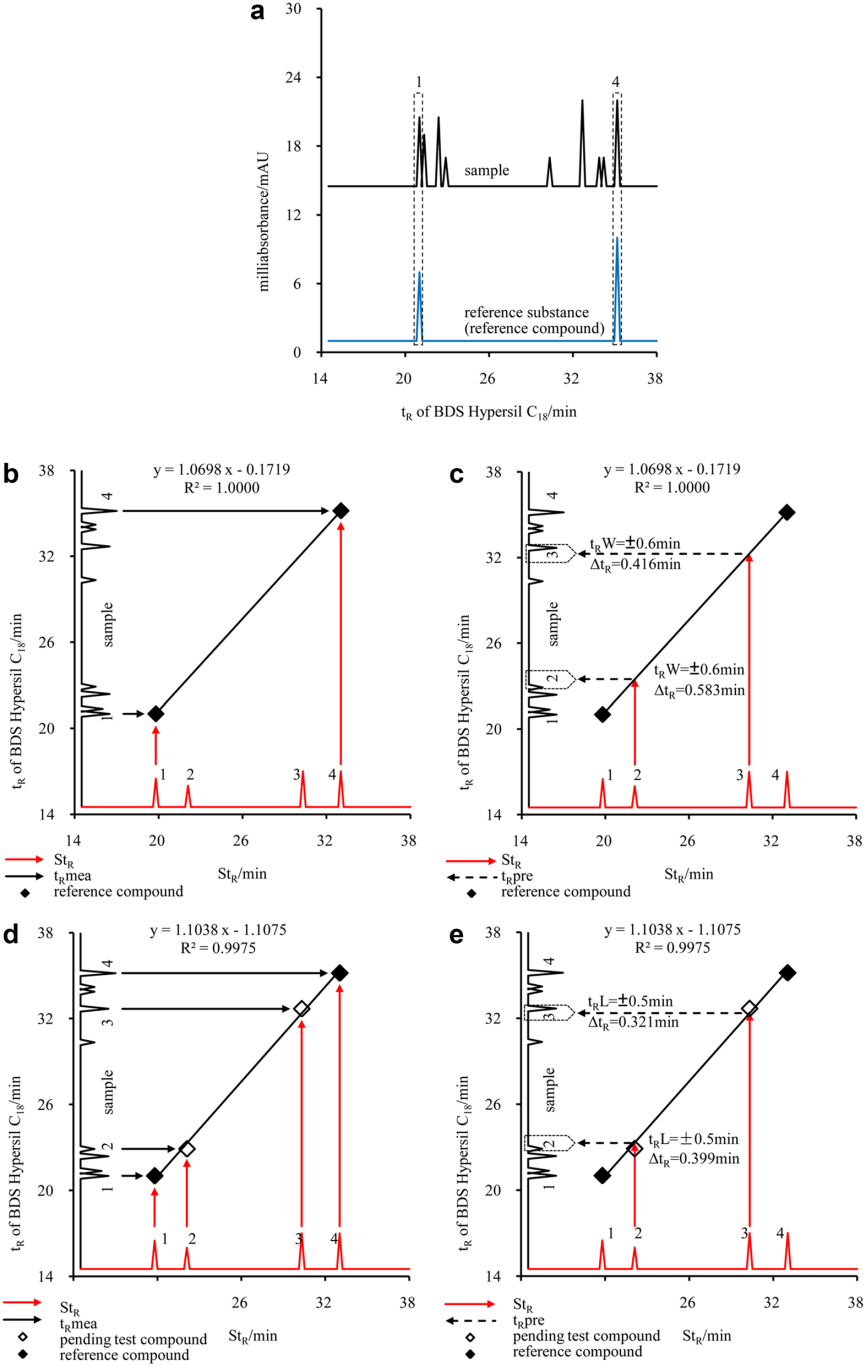
Flow chart of LCTRS (Paridis, code No. is the same as that in Fig. 1)

Take Paridis as an example. First of all, reference substances solution of two reference compounds (Chonglou saponin VII and Chonglou saponin I) and sample solution were performed on a C_18_ column (col4: BDS Hypersil C_18_). The t_R_mea (21.014 and 35.170 min) of two reference compounds in the sample solution were obtained by the reference substances solution (Fig. 4a). Then two points, Chonglou saponin VII (19.803, 21.014) and Chonglou saponin I (33.035, 35.170), could be determined in the coordinate using St_R_ as abscissa and t_R_mea as ordinate. Based on the two points, the following linear equation was given: y = 1.0698x − 0.1719 (Fig. 4b). Taking St_R_ of analytes (Chonglou saponin VI and Chonglou saponin II) into equation, the t_R_pre of Chonglou saponin VI (23.481 min) and Chonglou saponin II (32.263 min) were attained, respectively. Finally, in the chromatogram of the sample solution, the corresponding peaks of Chonglou saponin VI and Chonglou saponin II can be found within the range of t_R_pre ± t_R_W (t_R_W is abbreviation of t_R_ window, in this case is 0.6 min), as shown in Fig. 4c. It can be seen that Δt_R_ of analytes calculated by prediction of two points were 0.583 min and 0.416 min (The t_R_mea of analytes were 22.898 min and 32.679 min).

### Procedure of multiple points regression

After assignment of the peaks of analytes in the sample solution by prediction of two points regression, the t_R_mea of those peaks should be validated by multiple points regression. In this procedure, t_R_mea and St_R_ of reference compounds and analytes were used to fit a multiple points linear regression: Y = 1.1038x − 1.1075 (Fig. 4d). Taking St_R_ of analytes (Chonglou saponin VI and Chonglou saponin II) into new equation, the new t_R_pre of Chonglou saponin VI (23.297 min) and Chonglou saponin II (32.358 min) were calculated. If Δt_R_ of all analytes were less than the given t_R_L (t_R_L: t_R_ limit, in this case is 0.5 min), the prediction was success, otherwise failure (Fig. 4e). In this case, Δt_R_ were 0.399 and 0.321 min, respectively. The step of validation by multiple points was based on the principle of stepwise linear regression, which can further improve the prediction accuracy.

The purpose of setting that the t_R_W is larger than t_R_L is to increase the amount of suitable columns and to improve the accuracy of prediction. Generally, the recommended ranges of t_R_W and t_R_L are 0.8–2.0 and 0.5–1.5 min, respectively. If necessary, the values can be adjusted in accordance with different samples under different chromatographic conditions. If the Δt_R_ of some compounds are large, their t_R_W and t_R_L can be set individually.

### Sequential matching rule

**Fig. 5:**
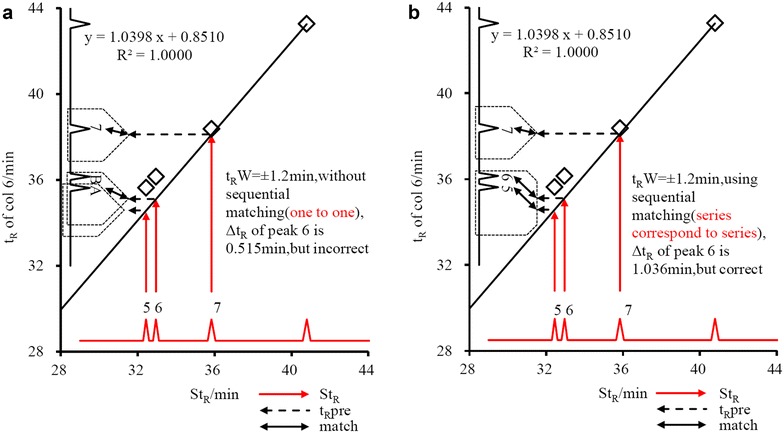
Advantage of sequential matching (Psoraleae, code No. is the same as that in Fig. 1)

If the t_R_ of two peaks are too close, e.g. less than 2 min, there would be a mistake for peak matching by the least Δt_R_ rule. Take Psoraleae for example, as shown in Fig. 5a, peak #6 was assigned to peak A in the sample solution on col6 (Kromasil C_18_) with a small Δt_R_ of 0.515 min. However, peak #5 was not found and peak B in the sample solution was not matched. When t_R_W was set as 1.2 min, t_R_mea of peak A was within the window of t_R_pre of peak #5. t_R_mea of the peaks A and B would both fall into the window of t_R_pre of peak #6. Because of the existence of one common peak (peak A), peaks #5 and 6 should be treated as peak series for sequential matching. That is, the earlier t_R_pre will be matched to the peak with the earlier t_R_mea. Although Δt_R_ of peak #6 increased to 1.036 min, the match results were correct, as shown in Fig. 5b. This rule can be further applied to multiple-peak series, which has a close t_R_.

### Comparison between LCTRS method and RR method

The comparison among unadjusted RR method, adjusted RR method (dead time was measured by ammonium nitrate as probe compound), prediction by two points, and validation by multiple points was summarized in Tables 4 and 5. The results showed that the unadjusted RR method and adjusted RR method were similar, their prediction accuracy were bigger and suitable for less positive columns. But the prediction deviation was reduced and the number of positive columns was increased by LCTRS method. The best was validation by multiple points which was based on the prediction by two points.

**Table 4:** Comparison result by four methods (Psoraleae)

Method	Maximum of Δt_R_/min	Average of Δt_R_/min	Number of positive columns^a^
Unadjusted RR	3.494	0.804	2
Adjusted RR	3.001	0.886	3
Prediction by two points	2.194	0.599	5
Validation by multiple points	1.689	0.465	9

^a^The columns which meet the following requirements are called positive column, (1) the resolution of peaks meets the requirements; (2) Δt_R_ of all pending test compounds are no more than t_R_L (for Psoraleae is 1.2 min)

**Table 5:** Comparison of prediction result in four methods (Paridis)

Method	Maximum of Δt_R_/min	Average of Δt_R_/min	Number of positive columns^a^
Unadjusted RR	1.811	0.420	12
Adjusted RR	1.562	0.410	9
Prediction by two points	0.836	0.283	25
Validation by multiple points	0.545	0.204	30

^a^t_R_L = 0.5 min

### Exclusion of column and compound by linear fitting

Nonlinear shift of t_R_ for a compound on different columns could be caused either by different column packing materials and use of other packing techniques, or by the different compound structure. In order to exclude the columns and compounds with relatively large nonlinear shift, linear fitting of t_R_mea and St_R_ were performed. The following rules were used to identify the outlier column and compound. (1) In a regression scatter plot, the compounds obviously deviated from a regression line (the correlation coefficient is usually less than 0.99). (2) Δt_R_ was usually larger than 1–2 min. The excluded columns and compounds would not be used for St_R_ calculating.

**Fig. 6:**
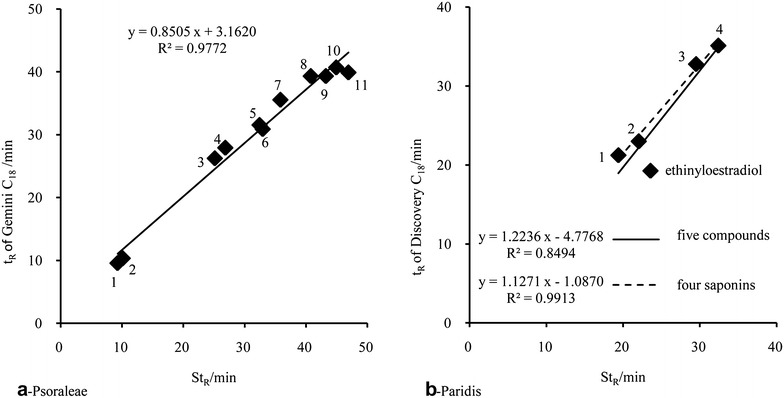
Outlier column (**a**) and Outlier compounds (**b**), code No. is the same as that in Fig. 1

For Psoraleae: no obvious nonlinear deviation was observed of all 11 compounds. 23 columns met the requirements (the average of correlation coefficient was 0.9989). The outlier columns were col2, 8, 12 (Fig. 6a), 16, 19, 20 and 30. For Paridis: no obvious nonlinear deviation was observed of four saponins. All 30 columns met the requirement with average correlation coefficient of 0.9993.

In order to simulate t_R_ of compound with large structural difference, reference substances solution of ethinylestradiol mixing with four Chonglou saponins were used to measure t_R_ of those five compounds on 30 columns. Nonlinear shift of ethinylestradiol was observed on col1 (Fig. 6b), 2–6, 8, 11, 15–18, 26–28 and 30. It appears that the HPLC retention behaviors of ethinylestradiol and four Chonglou saponins were significantly different on this chromatographic condition. It further indicated that the classification and similarity evaluation of columns should be based on the characteristics of columns as well as analytes.

If the outlier compounds cannot be excluded. The following approaches could be used: (1) specify one or more suitable columns; (2) provide reference substances for those compounds; (3) use UV–Vis spectrum and/or mass spectrum for assistant peak identification.

### Selection of two reference compounds

**Fig. 7:**
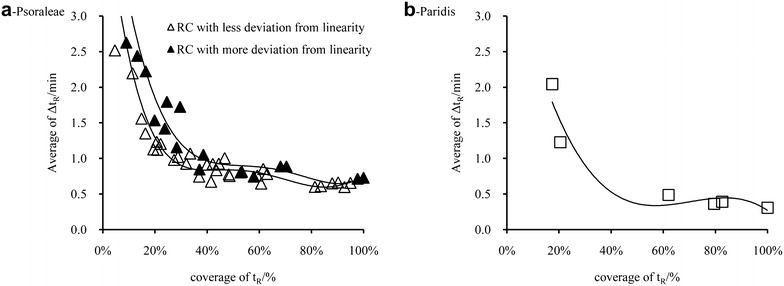
Selection of two reference compound (abbreviated as RC)

Ideally there should be no difference in selecting any of the two compounds as reference compounds. However, because of the difference of HPLC instruments, columns, compounds structure, complexity of elution condition, and accidental error of analysis, different selection of reference compound pairs will make differences. In order to find out the rule for reference compounds selection, each combination of possible reference compound pairs for the two medicines was studied. The average of Δt_R_ corresponding to each reference compound pair were calculated and shown in Fig. 7. It can be seen that, for the two medicines, the Δt_R_ of prediction by two points step would be decreased with increasing coverage of t_R_ [as shown in formula (5). The coverage of t_R_ is a reflex of the relative position of the two reference compounds. The first compound is at one end (with smaller t_R_), the last compound is at the other end (with bigger t_R_). If the coverage is high, the two reference compounds are near both ends, otherwise they are in the middle or near the same end]. The coverage corresponding to the smallest Δt_R_ was 80–100%.The results of Psoraleae (Fig. 7a) showed the advantage of choosing reference compounds with smaller linear deviation, when the coverages of t_R_ were similar. Therefore, the optimized reference compounds for Psoraleae were peak #2 and peak #8, rather than peak #1 and peak #11 which had a maximum coverage of t_R_ but with more deviation from linearity. The selection rule decreases the randomness of choosing reference compounds and the amount of calculation (or the Δt_R_ of all possible reference compound pairs will be calculated every time). The accurate and simple selection procedures were as follows. Firstly, Select two reference compound pairs with large t_R_ coverage (80–100%). Secondly, exclude compounds with large linear deviation based on the linear fitting results. Thirdly, calculate the Δt_R_ of the rest reference compound pairs and select reference compound pairs with the smallest Δt_R_.

5 $$ Coverage \; of \; t_{R} = \frac{{t_{R2} - t_{R1} }}{{t_{Rlast} - t_{Rfirst} }} $$

t_R2_ is t_R_ (or St_R_) of second reference compound; t_R1_ is t_R_ (or St_R_) of first reference compound; t_Rlast_ is t_R_ (or St_R_) of last compound; t_Rfirst_ is t_R_ (or St_R_) of first compound.

In summary, the establishment procedures of LCTRS were as follows. Firstly, select five–fifteen different brands of C_18_ columns, and record the HPLC chromatograms of reference substances and sample on all columns. Secondly, calculate initial St_R_ by using all columns, and perform linear fitting of t_R_ on each column with St_R_. Exclude outlier columns and compounds, and recalculate final St_R_ using remaining columns. Finally, select two reference compounds with large t_R_ coverage and low linear deviation.

## Discussion

### Advantages of LCTRS method

According to the study of Wang et al. [22], t_R_ of the compounds on different HPLC system follows the linearity principle. The RR method can be regarded as external standard one point method, which means the regression line is forced to pass origin. However, most of the linear equations have intercepts, which is why the deviation of unadjusted RR method was large. For considering the dead time, adjusted RR method should be better than unadjusted RR method in theory. But the probe compound for dead time measurement would be interacted with mobile phases and stationary phases of the columns. The interaction would increase the error in dead time measurement. So the prediction accuracy of this method was not improved in practice. For prediction by two points and validation by multiple points, dead time, gradient delay, volume exclusion effect of stationary phase, retention behavior of homologous compounds and so forth, were fully considered. Thus, the prediction accuracy was significantly improved. Stepwise linear regression was used in the validation by multiple points step, which further improved the prediction effect.

### Compatibility of LCTRS method and RR method

Both LCTRS method and RR method are equivalent in mathematics. Formulas can be expressed in the same form. In the LCTRS, calibrated retention (CR) is defined as the ratio of St_R_ of analytes to reference compounds, as shown in formula (6). Different from RR, CR is based on statistics of St_R_. Thus, its prediction accuracy was equal to LCTRS (only equal to prediction by two points).

6 $$ CR = RR = \frac{{t_{Ri} - t_{R1} }}{{t_{R2} - t_{R1} }} $$

where t_Ri_ is St_R_ of analytes in CR, or t_R_ of analytes in RR; t_R1_ is St_R_ of the first reference compound in CR, or dead time in adjusted RR, or zero in unadjusted RR; t_R2_ is St_R_ of the second reference compound in CR, or t_R_ of reference compound in RR.

## Conclusion

A new method for t_R_ prediction of HPLC chromatographic peaks was proposed. 16 compounds in two medicines under isocratic or gradient elution conditions were tested through three brands of HPLC instruments with 30 different brands of C_18_ columns. It is demonstrated that the method is simple, accurate, and robust for more HPLC columns. Furthermore, the calculation approach of St_R_ and the selection rule of the two reference compounds were discussed.

Both multi-components analysis in TCMs and determination of related substances in pharmaceutical chemicals need lots of reference substances for peak identification. But it may be not affordable for routine analysis and research using all reference substances. LCTRS is a simple and low-cost alternative method for peak identification. Compared with RR method, it need one more reference substance but is more accurate and suitable for more HPLC columns. LCTRS method provides a good prospective application for overall quality evaluation of TCMs and impurities analysis in pharmaceutical chemicals.

## Additional file

**Additional file 1.** PDF of reference 22. The authors have got the permission from the copyright holder to use the article.

**Additional file 2.** English translation of reference 22. The authors have got the permission from the copyright holder to use the article.

**Additional file 3.** The minimum standards of reporting checklist.

## Abbreviations

TCMs: traditional Chinese medicines
RR: relative retention
LCTRS: linear calibration using two reference substances
t_R_: retention time
SSDMC: single standard to determine multi-components
St_R_: arithmetic average of t_R_
Δt_R_: deviation of t_R_
t_R_pre: predicted retention time
t_R_mea: measured retention time
CR: calibrated retention
