# Supplementary material for: A simple method for HPLC retention time prediction: linear calibration using two reference substances
Source: Chin Med. 2017 Jun 19;12:16. doi: 10.1186/s13020-017-0137-x (PMC5477150; doi:10.1186/s13020-017-0137-x)
Supplement: Supplementary file 1 — Additional file 1. PDF of reference 22. The authors have got the permission from the copyright holder to use the article. [file 13020_2017_137_MOESM1_ESM.pdf]

一种提高色谱指纹谱保留时间重现性的新方法

王龙星　肖红斌　梁鑫淼*
（中国科学院大连化学物理研究所，大连 116012）

摘　要　通过色谱热力学分析发现，在相同的分析条件下，即使采用不同的液相色谱系统或不同的色谱柱，组分的保留时间存在简单的线性关系，应用该线性关系可提高不同反相 C_{18} 柱间保留时间重现性，经过实际样品在不同操作条件下的验证，表明该方法是正确而可行的。

关键词　指纹谱，保留时间重现性，高效液相色谱

1　引　　言

色谱指纹谱是近年来中药质量控制的热点[1,2]，提高不同实验室间色谱数据的重现性无疑对指纹谱质量控制具有重要的意义。反相 C_{18} 柱是高效液相色谱中使用最广泛的色谱柱，提高不同反相 C_{18} 柱间保留时间的重现性对指纹谱的实际应用有较大的帮助。对使用反相 C_{18} 柱的液相色谱分析而言，造成在同一条件下的保留时间漂移的原因是液相色谱仪器系统引起的保留时间漂移和色谱柱的差异引起的保留时间漂移。本文通过色谱热力学理论分析找到了一个新方法，能够有效地提高不同 C_{18} 柱保留时间的重现性，对两个不同的 C_{18} 柱校正前后保留时间的差别从最大 12 min 减少到了一般在 0.2 min 以内。

2　理论部分

根据色谱热力学[3]，组分的容量因子 k 与色谱两相分配过程中的焓变 ΔH 与熵变 ΔS 的关系为：（β 是相比）

$$\ln k = \ln \frac{\Delta S}{R} - \frac{\Delta H}{RT} - \ln\beta \quad (1)$$

由于使用的填料类型是相同的，我们认为，在相同的操作条件下不同 C_{18} 柱的焓变 ΔH 与熵变 ΔS 是相同的。它们的差别主要是：不同 C_{18} 柱其填料的相比 β 是有一定差别，不仅不同品牌填料间的 β 不同，而且随着色谱柱的使用，固定相有轻微的流失这也将带来相比的变化；由装柱过程带来的填料间孔隙的大小与几何性状的不同以及色谱柱本身长度的不同引起的不同 C_{18} 柱间死时间的差别。

基于上面的分析与式(1)可以推出式(2)和式(3)（k_1、k_2 是两根不同 C_{18} 柱的保留因子，β_1、β_2 是它们的相比）。

$$k_1 = \exp(\ln \frac{\Delta S}{R} - \frac{\Delta H}{RT}) \times \frac{1}{\beta_1} \quad (2)$$

$$k_2 = \exp(\ln \frac{\Delta S}{R} - \frac{\Delta H}{RT}) \times \frac{1}{\beta_2} \quad (3)$$

式(2)除以式(3)得式(4)。式(4)表明不同 C_{18} 柱在同一操作条件下的保留因子为简单的正比关系。

$$\frac{k_1}{k_2} = \frac{\beta_2}{\beta_1} \quad (4)$$

$$V_1 = \frac{L_1}{t_{m1}(1 + k_1)} \quad (5)$$

$$V_2 = \frac{L_2}{t_{m2}(1 + k_2)} \quad (6)$$

2002-11-14 收稿；2003-04-07 接受
本文系国家 973 计划资助项目(No.G1999054406)

?1994-2015 China Academic Journal Electronic Publishing House. All rights reserved.　http://www.cnki.net

式(5)和式(6)是两柱上溶质谱带的运动速度与保留因子的关系(V_1、V_2 是谱带在两柱上的运动速度, L_1、L_2 是两柱间的柱长)。式(5)、式(6)两式都是简单线性函数, 而 k_1、k_2 又成正比, 根据代数基本原理[4], 谱带在两柱上的运动速度应当为线性关系。这也就是说在同一液相色谱系统上, 组分在两根色谱柱上的保留时间为简单线性关系。式(7)列出了这种线性关系, t_1、t_2 是两柱的保留时间 a, b 是常数。

$$t_{r2} = a + b \times t_{r1} \tag{7}$$

对现代液相色谱仪器来说, 流速及流动相比例的控制精度越来越高, 一般认为[5,6] 由液相色谱仪器引起的保留时间漂移可以忽略。对于等度分析这是正确的, 但对梯度分离情况就复杂了。图 1 是二元梯度液相色谱系统的简单示意图。流动相比例是电磁阀的切换频率决定的。当电磁阀的切换频率发生变化时, 柱头的流动相配比不是立即变化, 而是有一段时间的延迟。这个延迟时间受多种因素的影响, 如泵结构, 连接管线的长度等。即使对同一厂家的液相色谱系统, 这个梯度延迟也是不相等的。我们的实验室拥有两套 Alliance 2690(Waters 公司)液相色谱系统, 经测量它们的梯度延迟分别是 0.9 及 0.6 min。对采用线性梯度的液相色谱分离过程, 梯度延迟对保留时间的影响[7,8] 见式(8)。

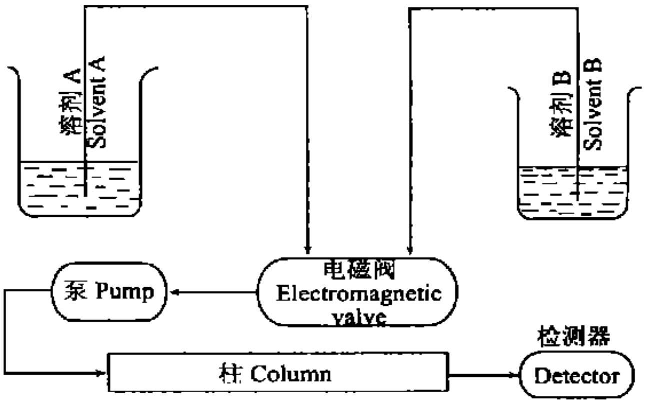

图 1　二元梯度液相色谱系统示意图

Fig. 1　Simple demo graph of a binary gradient high performance liquid chromatographic (HPLC) system

$$t_r = (t_g / S\Delta\Phi)\ln[S\Delta\Phi t_0 k_0 \exp(-S\Phi_i)/t_g + 1] + t_0 + t_d \tag{8}$$

t_r 是保留时间, t_g 为梯度时间, $\Delta\Phi$ 为流动相组成的变化, t_0 为死时间, Φ_i 为初始流动相组成, t_d 是系统梯度延迟时间, S 和 k_0 是常数。式(8)说明在相同的条件下, 同一根色谱柱在不同液相色谱系统上的保留时间也呈线性关系。

综合上述分析可以得到这样一个结论: 无论是等度分离还是梯度分离, 在相同的分析条件下, 即使采用不同的液相色谱系统, 并使用不同的色谱柱, 组分的保留时间存在简单的线性关系。在实际中, 可以应用这种线性关系, 提高保留时间的重现性。首先在相同的操作条件下, 用一合适的含 5~9 个组分的标准样品, 分别测定其在两个实验室的保留时间, 利用这组保留时间进行线性回归可获得常数 a、b 的数值。然后我们用获得的 a、b 对其它的保留时间进行校正, 校正后, 使保留时间的重现性将大大提高。本文即利用这种线性关系对 2 根不同 C_{18} 柱在 5 个不同梯度、等度条件的 9 个组分的保留时间进行了校准, 取得了较好的效果。

3　实验部分

实验使用的试剂硝基苯(NB)、苯甲酸乙酯(EBT)、氯苯(CB)、邻二甲苯(OX)、对二甲苯(PX)、乙苯(EB)、苯甲酸丁酯(BBT)、邻二乙苯(ODB)及丁苯(BB)均为分析纯, 用来配制样品。Waters Alliance 2690 液相色谱系统, Waters 996 二极管阵列检测器。色谱纯乙腈及 Millipore 超纯水作为流动相。硝酸钠用于测定死时间, 本实验使用了如下两根 C_{18} 柱: (1) Column A, Hypersil ODS-2 4.6 mm × 250 mm (New); (2) Column B, Hypersil ODS-2 4.6 mm × 250 mm (Have been used for one year)。

在实验中使用了两套 Alliance 2690(Waters 公司)液相色谱系统, 简记为 System A, B。表 1 列出了本文使用的 5 个不同的等度或梯度条件。

4　结果与讨论

4.1　验证不同 C_{18} 柱间保留因子间的正比关系

?1994-2015 China Academic Journal Electronic Publishing House. All rights reserved.　http://www.cnki.net

图2显示了柱A与柱B上9个样品在3个不同等度条件下保留因子的关系。从图中我们可以看到,这27个不同条件下数据点间有良好的线性关系,相关系数达到0.9998,并通过了原点。这张图说明了本文理论部分推出的不同C_{18}柱在同一操作条件下的保留因子成正比的结论是正确的。

表1　实验采用的5个梯度或等度条件

Table 1　The five different isocrotic or gradient conditions in the experiments

	条件1 Condition 1	条件2 Condition 2	条件3 Condition 3	条件4 Condition 4	条件5 Condition 5
操作模式 Operation mode	等度 Isocratic	等度 Isocratic	等度 Isocratic	梯度 Gradient	梯度 Gradient
流速 Flow rate(mL/min)	1.0	1.0	1.0	1.0	1.0
初始乙腈浓度 Initial concentration of acetonitrile	45%	55%	65%	10%	25%
初始保持时间 Initial hold time(min)				5	1
梯度斜率 Ramp				2%/min	1.5%/min
终止乙腈浓度 Final concentration of acetonitrile				100%	100%
终止保持时间 Final hold time				8 min	10 min

4.2　验证不同C_{18}柱间保留时间的线性关系

表2列出了柱A与柱B在相同或不同液相色谱系统及5个不同等度或梯度条件下保留时间的线性相关系数,所有的线性相关系数都大于0.9993。图3列出了不同色谱柱及不同液相色谱系统下保留时间的线性回归曲线。这些数据表明了无论是使用梯度或等度条件,不同C_{18}柱在相同操作条件下的保留时间呈良好的线性关系。

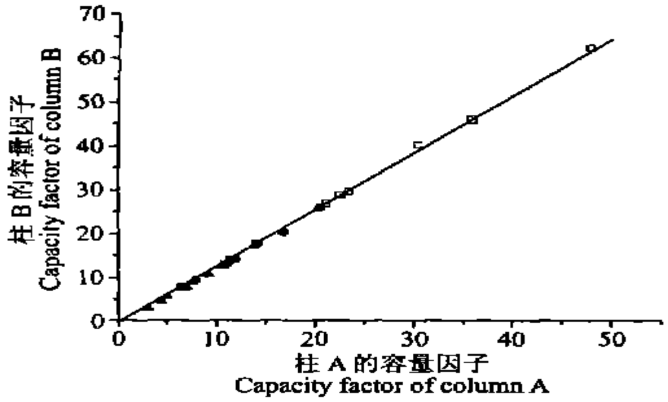

图2　两根不同C_{18}柱A及B上的容量因子间的关系图

Fig.2　The relationship between retention factors of two different C_{18} column A and B

(□)条件3(condition 3)(●)条件4(condition 4)(▲)条件5(condition 5),$r=0.9998$。

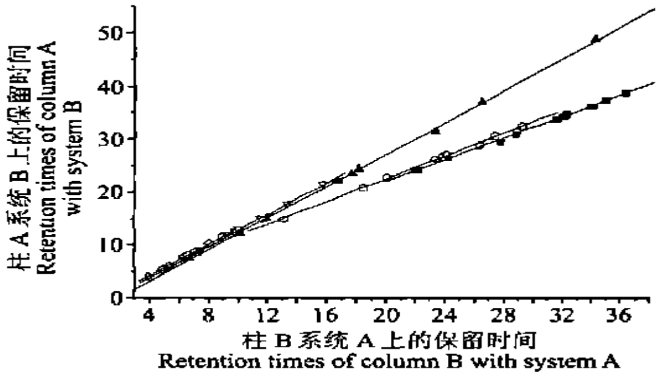

图3　在不同的液相色谱系统上不同C_{18}柱保留时间的关系图

Fig.3　Plots of retention times on different columns and with different HPLC systems

(■)条件1(condition 1);(○)条件2(condition 2);(▲)条件3(condition 3);(▽)条件4(condition 4);(◇)条件5(condition 5)。

表2　不同色谱柱上同一操作条件下保留时间的线性回归系数

Table 2　The linear regression coefficients of retention times on column A and B under the same condition

	柱A系统A对柱B系统A Column A on system A versus column B on system A	柱A系统B对柱B系统A Column A on system B versus column B on system A
条件1 Condition 1	0.9996	0.9993
条件2 Condition 2	0.9999	0.9996
条件3 Condition 3	0.9997	0.9997
条件4 Condition 4	0.9995	0.9997
条件5 Condition 5	0.9994	0.9995

4.3　不同C_{18}柱间保留时间校正前后绝对误差的对比

表3列出了不同色谱柱及色谱系统下,5个不同梯度条件下的保留时间。表4列出了在5个不同条

?1994-2015 China Academic Journal Electronic Publishing House. All rights reserved.　http://www.cnki.net

件下的保留时间校正前后的绝对误差，从中可以看到柱 A-System A 与柱 B-System B 在校正前保留时间的绝对误差较大，最大 15 min；经过线性校正后的保留时间绝对误差变的较小，一般在 0.2 min 以内，最大不超过 0.7 min。保留时间的绝对误差在校正后也得到了较大改善，进行线性校正的效果是很好的。

表 3　在不同色谱柱及液相色谱系统下获得的保留时间(min)

Table 3　Retention times under five different conditions on column A and B with different HPLC systems (min)

		NB	EBT	CB	OX	PX	EB	BBT	ODB	BB
系统 A 柱 A<br>System A column A	条件 1 Condition 1	23.763	29.269	30.699	33.623	34.029	34.304	35.774	37.029	38.438
	条件 2 Condition 2	14.611	20.401	22.299	25.887	26.420	26.745	28.679	30.235	32.128
	条件 3 Condition 3	7.464	12.243	15.102	22.104	23.534	24.348	31.398	36.950	48.912
	条件 4 Condition 4	5.260	7.505	8.895	11.908	12.390	12.837	14.857	17.708	21.441
	条件 5 Condition 5	4.124	5.339	6.075	7.563	7.732	8.002	8.659	10.222	11.575
系统 A 柱 B<br>Bsystem A column B	条件 1 Condition 1	22.011	27.647	28.734	31.508	31.932	32.147	33.866	34.772	36.183
	条件 2 Condition 2	13.075	18.435	19.966	23.235	23.800	24.041	26.209	27.329	29.202
	条件 3 Condition 3	6.687	10.120	11.900	16.671	17.668	18.146	23.266	26.429	34.151
	条件 4 Condition 4	4.902	6.510	7.426	9.447	9.779	10.069	11.585	13.287	15.693
	条件 5 Condition 5	3.962	4.829	5.316	6.411	6.420	6.598	7.110	8.061	8.930
系统 B 柱 A<br>System B column A	条件 1 Condition 1	24.296	29.606	31.093	34.019	34.441	34.712	36.200	37.465	38.847
	条件 2 Condition 2	15.005	20.916	22.743	26.319	26.833	27.163	29.066	30.644	32.499
	条件 3 Condition 3	7.520	12.289	15.137	22.125	23.559	24.386	31.425	36.978	48.981
	条件 4 Condition 4	5.302	7.531	8.915	11.928	12.429	12.884	14.906	17.758	21.491
	条件 5 Condition 5	4.152	5.392	6.118	7.601	7.755	8.052	8.718	10.282	11.621

NB: nitrobenzene; EBT: ethylbenzoate; CB: chlorobenzene; OX: o-xylene; PX: p-xylene; EB: ethylbenzene; BBT: butylbenzoat; ODB: o-diethylbenzene; BB: butylbenzene。

4.4　本方法与单内标法的对比

为减少保留时间的漂移，在液相色谱分析中往往加入一个内标化合物，以相对保留时间代替保留时间。这样作虽然也可以减少不同 C_{18} 柱保留值的差异，但是效果并不理想。从表 3 和表 4 的数据可以看到，随着保留时间数值的增加，不同 C_{18} 柱的保留时间绝对误差也在增加。内标法只能校正保留时间与内标的保留时间差别较小的色谱峰，对有较多色谱峰的复杂样品来说，单一内标是无效的，并且内标法增加了样品处理的复杂性。而本文提出的方法只需要在测试实际样品前运行一次标准样品，并且对全部保留时间范围内的色谱峰的校正效果都较好，因此可以说本法更加简便有效。

表 4　校正前后不同色谱柱上保留时间绝对误差的对比(min)

Table 4　The compare of absolute errors of retention time before and after calibration on column A and B (min)

			NB	EBT	CB	OX	PX	EB	BBT	ODB	BB
柱 A 系统 A 对柱 B 系统 A<br>Column A system A versus column B system A	条件 1<br>Condition 1	Error(B)	1.752	1.622	1.965	2.115	2.097	2.157	1.908	2.257	2.255
		Error (A)	0.100	0.246	0.041	0.076	0.042	0.091	0.216	0.084	0.027
	条件 2<br>Condition 2	Error(B)	1.536	1.966	2.333	2.652	2.620	2.704	2.470	2.906	2.926
		Error(A)	0.065	0.100	0.114	0.145	0.070	0.128	0.261	0.050	0.082
	条件 3<br>Condition 3	Error(B)	0.777	2.123	3.202	5.433	5.866	6.202	8.132	10.521	14.761
		Error(A)	0.230	0.032	0.086	0.038	0.086	0.024	0.465	0.056	0.273
	条件 4<br>Condition 4	Error(B)	0.358	0.995	1.469	2.461	2.611	2.768	3.272	4.421	5.748
		Error(A)	0.099	0.011	0.001	0.009	0.019	0.011	0.179	0.022	0.108
	条件 5<br>Condition 5	Error(B)	0.162	0.510	0.759	1.152	1.312	1.404	1.549	2.161	2.645
		Error(A)	0.060	0.003	0.006	0.098	0.0096	0.008	0.066	0.024	0.056
柱 A 系统 B 对柱 B 系统 A<br>column A system B versus column B system A	条件 1<br>Condition 1	Error(B)	2.285	2.059	2.359	2.511	2.509	2.565	2.334	2.693	2.664
		Error(A)	0.170	0.355	0.007	0.061	0.044	0.093	0.199	0.128	0.050
	条件 2<br>Condition 2	Error(B)	1.930	2.481	2.777	3.084	3.033	3.122	2.857	3.315	3.297
		Error(A)	0.140	0.034	0.136	0.172	0.074	0.143	0.302	0.063	0.110
	条件 3<br>Condition 3	Error(B)	0.833	2.169	3.237	5.454	5.891	6.240	8.159	10.549	14.830
		Error(A)	0.356	0.046	0.122	0.076	0.144	0.037	0.709	0.080	0.453
	条件 4<br>Condition 4	Error(B)	0.400	1.021	1.489	2.481	2.650	2.815	3.321	4.471	5.798
		Error(A)	0.158	0.024	0.14	0.031	0.028	0.008	0.259	0.041	0.166
	条件 5<br>Condition 5	Error(B)	0.190	0.563	0.802	1.190	1.335	1.454	1.608	2.221	2.691
		Error(A)	0.079	0.016	0.010	0.153	0.012	0.017	0.086	0.049	0.082

Error(B)：校正前的保留时间绝对误差(the absolute error of retention time on different columns before calibration)；Error (A)：校正后的保留时间绝对误差(the absolute error of retention time on different columns after calibration)；NB, EBT, CB, OX, PX, EB, BBT, ODB and BB are same as in table 3)

4.5　本方法的适用范围

本文在色谱热力学的基础上证明了在同样分析条件下，不同 C_{18} 柱在不同液相色谱系统上的保留时

?1994-2015 China Academic Journal Electronic Publishing House. All rights reserved.　http://www.cnki.net

间为线性关系。本文的证明是建立在平衡分配机理的基础上。由于填料上残余硅羟基及金属离子的存在,对有的组分来说(如有机酸),吸附机理会变得不可忽略[9]。这种情况下,本文的结论会失效。但是在反相液相色谱中出现明显吸附的时候,柱效和保留时间的重复性都将严重降低。遇到这种情况,分析工作者往往在流动相内加入醋酸、磷酸等成分抑制组分的吸附现象,改善峰型与分离,在此情况下分配机理又将占主导地位。换句话说,绝大多数实际的分析过程都符合分配模型,因此,本文的结论在绝大多数情况下是成立的。

References

1 Xie Peishan(谢培山). *Chinese Traditional Patent Medicine*(中成药), **2000**, 22(6): 391～395

2 Ren Dequan(任德权). *Journal of Chinese Medicinal Materials*(中药材), **2001**, 24(4): 235～239

3 Lu Peizhang(卢佩章), Dai Chaozheng(戴朝正), Zhang Xiangmin(张祥民). *Basic Theory of Chromatography* (*Second Edition*)(色谱理论基础). Beijing (北京): Science Press(科学出版社), **1997**: 280～282

4 Alexander L(苏联: 亚里山大著). *Contents, Methods and Meaning of Mathematics*(数学: 它的内容、方法和意义). Beijing (北京): Science Press(科学出版社), **1962**: 217～281(Vol. 3)

5 Felinger A, Kele M, Guiochon G. *J. Chromatogr. A*, **2001**, 913: 23～48

6 Kele M, Guiochon G. *J. Chromatogr. A*, **2001**, 913: 89～112

7 Snyder L R, Dolan J W. *J. Chromatogr. A*, **1996**, 721, 3～14

8 Herman D P, Billet H A H, Galan L D. *J. Chromatogr. A*, **1989**, 463: 1～20

9 Steffeck R J. *LC GC North America*, **1995**, 13: 720～731

A New Method to Improve the Reproducibility of Retention Time on Reversed Phase C_{18} Columns in Different Laboratories

Wang Longxing, Xiao Hongbin, Liang Xinmiao*

(*Dalian Institute of Chemical Physics, Chinese Academy of Sciences, Dalian* 116012)

Abstract A great deal of high performance liguid chromatographic (HPLC) fingerprints of complex samples which are very helpful for chromatographer have been accumulated. However, the drifting of retention values on different laboratories retarded us to use these data effectively and sufficiently. The aim of this paper is to improve the reproducibility of retention times on different C_{18} columns with different HPLC systems. On the basis of the thermodynamic theory of HPLC, a new method is presented to improve the reproducibility of retention times on reversed phase C_{18} columns in different laboratories. Retention values of nine solutes on two different C_{18} columns under five different conditions were used to verify this method. The results show that this method is a good way to reduce the absolute error of retention times on different HPLC systems with different C_{18} columns.

Keywords Chromatographic fingerprint, retention time reproducibility, high performance liquid chromatography,

(Received 14 November 2002; accepted 7 April 2003)

?1994-2015 China Academic Journal Electronic Publishing House. All rights reserved. http://www.cnki.net
