# Supplementary material for: A simple method for HPLC retention time prediction: linear calibration using two reference substances
Source: Chin Med. 2017 Jun 19;12:16. doi: 10.1186/s13020-017-0137-x (PMC5477150; doi:10.1186/s13020-017-0137-x)
Supplement: Supplementary file 2 — Additional file 2. English translation of reference 22. The authors have got the permission from the copyright holder to use the article. [file 13020_2017_137_MOESM2_ESM.doc]

**2 The part of theory**

According to the thermodynamic theory of HPLC, ΔH is the enthalpy change and ΔS is the entropy change in the process of the chromatographic two-phase distribution. The relationship between capacity factor (k), ΔH andΔS is shown in formula (1), in which β represents phase ratio.

$\ln k=\ln\frac{\Delta S}{R}-\frac{\Delta H}{RT}-\ln\beta$ (1)

Different C18 columns are filled with the same type of packing material, so ΔH and ΔS are considered to be constant with the same chromatographic condition. The main differences among different C18 columns inculding: phase ratio (β) of packing materials in different C18 columns may be different, different brands of packing materials usually have different value of β, and as the consumption of chromatographic column, there is a slight loss of stationary phase, which might lead to changes in phase ratio; difference in dead time of all kinds of C18 columns is due to difference in pore sizes and geometrical properties of packing materials in the process of column packing and difference in column length.

Based on the above analysis and formula (1), formula (2) and formula (3) are deduced (k_1_ and k_2_ are capacity factors of two different columns, β_1_ and β_2_ are the phase ratios).

$k_{1}=\exp\left(\ln\frac{\Delta S}{R}-\frac{\Delta H}{RT}\right)\times\frac{1}{\beta_{1}}$ (2)

$k_{2}=\exp\left(\ln\frac{\Delta S}{R}-\frac{\Delta H}{RT}\right)\times\frac{1}{\beta_{2}}$ (3)

Formula (2) divided by formula (3) to obtain formula (4). Formula (4) indicates that there is a direct proportionality between capacity factors on different C18 columns under the same chromatographic condition.

$\frac{k_{1}}{k_{2}}=\frac{\text{β}_{2}}{\text{β}_{1}}$ (4)

$V_{1}=\frac{L_{1}}{t_{m1}\left(1+k_{1}\right)}$ (5)

$V_{2}=\frac{L_{2}}{t_{m2}\left(1+k_{2}\right)}$ (6)

Formula (5) and formula (6) explains the relationship between movement speed of solute band on two columns and the capactiy factor (V_1_ and V_2_ are velocity of solute band on two columns, L_1_ and L_2_ are the length of column ). Formula (5) and formula (6) are both simple linear function and k_1_ and k_2_ is in the direct ratio. Then according to the basic principle of algebra, velocity of solute band on two columns is nearly linear relationship. That is to say, retention times of components on two columns are linear relationship with the same chromatographic system. Formula (7) provides the linear relationship, t_1_ and t_2_ are retention times on two columns, a and b are constant.

$t_{r2}=a+b\times t_{r1}$ (7)

For modern liquid chromatograph, precision of speed and mobile phase ratio is higher and higher. It is generally recognized that drift of retention time caused by liquid chromatograph can be ignored. This is true for isocratic analysis, but the analysis on gradient is complex. Mobile phase ratio is controlled by switch frequence of magnetic valve. When the switch frequence of magnetic valve changes, mobile phase ratio on column cap can not change immediately. There is usually some time delay on the changement of mobile phase ratio. Pump structure, length of connecting line and other elements can affect the delay time. The delay time is unequal within the same brand chromatographic systems. For the liquid chromatographic separation process using linear gradient, formula (8) indicates the influence of gradient delay on retention time:

$t_{r}=\left(t_{g}/S\Delta\Phi\right)\ln\left[S\Delta\Phi_{t_{0}}k_{0}\exp\left(-S\Phi_{i}\right)/t_{g}+1\right]+t_{0}+t_{d}$ (8)

in which t_r_ is the retention time, t_g_ is the gradient elution time, ΔΦ is the change of mobile phase, t_0_ is the dead time, Φ_i_ is the original mobile phase, t_d_ is the delay time of system, S and k_0_ are constant.

Formula (8) indicates that for the same chromatographic condition, retention time on the same column with different chromatographic systems is linear relationship.

We can get that there is a linear relationship between the t_R_ of the compounds in different HPLC systems (including chromatographs and columns).
